# Bridging the Gap Between Believing and Memory Functions

**DOI:** 10.5964/ejop.7461

**Published:** 2023-02-28

**Authors:** Rüdiger J. Seitz, Hans-Ferdinand Angel, Raymond F. Paloutzian

**Affiliations:** 1 Medical Faculty, Heinrich-Heine-University Düsseldorf, Düsseldorf, Germany; 2 Karl Franzens University Graz, Graz, Austria; 3 Westmont College, Santa Barbara, CA, USA; University of South Wales, Cardiff, United Kingdom

**Keywords:** believing, brain, meaning, neural processes, memory, credition, beliefs

## Abstract

Believing has recently been recognized as a fundamental brain function linking a person’s experience with his or her attitude, actions and predictions. In general, believing results from the integration of ambient information with emotions and can be reinforced or modulated in a probabilistic fashion by new experiences. Although these processes occur in the subliminal realm, humans can become aware of what they believe and express it verbally. We explain how believing is interwoven with memory functions in a multifaceted fashion. Linking the typically rapid and adequate reactions of a subject to what he/she believes is enabled by working memory. Perceptions are stored in episodic memory as beneficial or aversive events, while the corresponding verbal descriptions of what somebody believes are stored in semantic memory. After recall from memory of what someone believes, personally relevant information can be communicated to other people. Thus, memory is essential for maintaining what people believe.

Living beings are constantly exposed to ambient information in their environment. While objects appear stable, events are perceived as a change in the environment with a beginning and an end (Zacks & Tversky, 2001). Since the often rapidly changing information in the environment has effects on the physical integrity, prosperity, and ultimately on the survival, living beings have to classify the information with respect to its most likely subjective relevance. In systems neuroscience terms, the information perceived from the external world is integrated with an internal emotional state into personally meaningful probabilistic representations that determine a person’s attitude, actions and predictions (Seitz et al., 2018). Accordingly, perception and valuation are the fundamental neural processes mediating what someone believes at a given point in time. Importantly, by the inherent capability to provide predictions about future events, believing can constrain behavior in a phylogenetically advantageous fashion (Connors & Halligan, 2020; Seitz et al., 2018). Accordingly, believing can be assumed to exert a tremendous influence on a person’s behavior, since it links his/her prior experience with his/her prospective acts.

Recently, the concept of credition (a neologism derived from Latin credere, to believe) has been presented (Angel et al., 2017). The concept posits that believing constitutes an assembly of brain functions similarly to cognition and emotion (Angel & Seitz, 2016). The focus of credition on brain functions provides an innovative perspective on debates which are concerned with the long-standing arguments about beliefs as entities separate from knowledge but highly related to the notion of truth (Leitgeb, 2017). Considering the formation of what one believes and its updating in response to new experiences avoids these theory-based issues but rather opens the perspective to empirical studies on the neural processes underlying believing and the resulting behavior.

As described earlier, most of what people believe manifests spontaneously on a pre-linguistic level (Seitz et al., 2018). Due to the rapidly changing percepts a subject typically is not aware of such beliefs that, therefore, have been labeled primal beliefs or belief precursors (Oakley & Halligan, 2017). However, after shorter or longer periods of time a person can become aware of what he/she is believing. Then, he/she recalls from memory his/her prior perception together with its associated personal meaning. Also, the person will compare his/her past predictions of future events with the actual event. Thus, the person will recall when and where the perception happened before and what it meant to him/her. These processes render the person consciously aware of the content of a given belief that thereby can be stated verbally and also communicated to other people (Oakley & Halligan, 2017; Seitz & Angel, 2020). Consequently, one can hypothesize that there may be an important interaction of believing and memory functions. Notably, this relation has not been addressed so far and, therefore, deserves further exploration. Here, we will outline how memory functions support the processes of believing. We will demonstrate two novel aspects, namely:

(a) that the processes of believing are tightly linked to encoding of subjectively relevant ambient information in memory.

(b) that the retrieval of this information encoded in memory plays an important role in how a person becomes aware of and semantically codes what he/she is believing.

Thus, we will show that there is no gap between memory and believing functions but that they are tightly interrelated.

## Neural Processes of Believing

**Figure 1:**
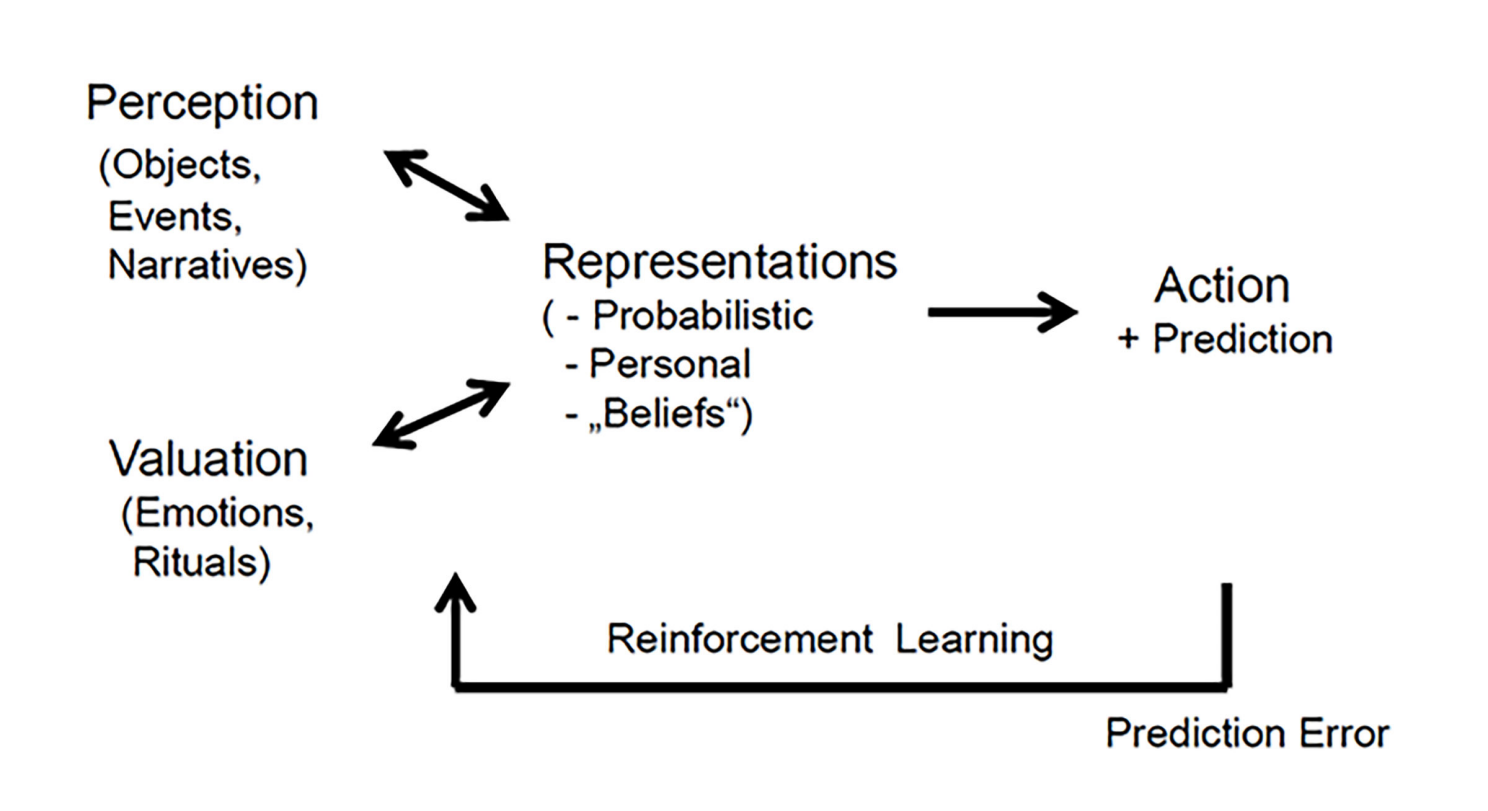
Schematic Model of the Neural Processes Affording Formation and Updating of Beliefs *Note*. Shown are the different types of ingoing information leading to corresponding categories of beliefs. The bi-directional arrows indicate bottom-up and top-down processing. Prediction errors of actions are fed backwards allowing for belief updating.

Let us begin by explaining the relation between believing and beliefs. Information from the environment undergoes re-iterative bottom-up and top-down processing in dedicated sensory brain systems resulting in a supraordinate meaning of the perceived items, including their attributed emotional value (Connors & Halligan, 2015; Seitz et al., 2018), as illustrated in Figure 1. Objects and events are evaluated and processed in a multisensory manner with the ability to discriminate object representations as early as 100 ms. after the onset of stimulation (Matusz et al., 2017). This underscores that processing in the nervous system occurs rapidly such that most information processed by the brain will not reach conscious awareness and initially is not coded verbally (Baumgartner et al., 2018). But signals of specific emotions or emotional strength may enhance neural processing efficiency and competitive strength of significant events (Pourtois et al., 2013). Moreover, the rapid and efficient selection of emotionally salient or goal-relevant stimuli in the environment is crucial for flexible and adaptive behavior (Peil, 2014). Accordingly, information processing in the brain is held to result in probabilistic accounts of more or less ambiguous information that have been called “priors” or “sensory precursors” (Connors & Halligan, 2020). These accounts are stored as imaginations with strong personal meaning in the medial parietal cortex (precuneus) that has been labeled metaphorically as the mind’s eye (Fletcher et al., 1995). This corresponds to the concept of unconscious knowledge, which is fundamental for an understanding of human thought and mentation (Augusto, 2010). Similarly, Taves and Asprem (2017) speak of a subject’s event knowledge. Importantly, however, these accounts or neural representations are not neutral but, in addition, also convey personal meaning that renders them behaviorally relevant and allows the person to act instantaneously and adequately. However, the number of processing repetitions required for meaning making may be modulated dynamically for different items and occasions.

Three different categories of information in the human environment have been differentiated (Seitz & Angel, 2020). The first category concerns concrete, physical objects. They are valuated in terms of emotional loading including aesthetic value, desirability or averseness, and threat (Ishizu & Zeki, 2013; Rolls, 2006; Thiruchselvam et al., 2016). The second category concerns the relations of a person to objects or to other people in the constantly changing environment. Processing of these two categories of ambient information results in the formation of so-called primal beliefs. They reflect the perceptive capabilities of humans, do not depend on language and manifest below the level of conscious awareness. They are of great evolutionary importance because they allow humans to react appropriately to challenging organisms, objects, and events as well as to interact socially with other individuals. Information of the third category refers to narratives that emerge from higher order conceptual processing requiring the use of a symbolic language. During evolution humans have been used to telling stories about their own and other people’s past, their origins, and their goals, and their future after physical death (Belzen, 2010). The resulting abstract, so-called conceptual beliefs pertain to ecological, social, cultural, religious, and political contents—all of which provide differentiated sources of identity, normative rules, and affective attachments (Brandt et al., 2019). They provide the contextual information for ritual acts that are strong affective enhancers of prosocial behavior (Hobson et al., 2018).

Accordingly, beliefs which are labels for the result of the neural processes affording believing. These processes render them omnipresent and indispensable in human life. They are suited to afford decision making concerning selection of actions and prediction of future events by optimization (Friston, 2010). The information held online as beliefs allows us to select actions with respect to active inference and predictive coding. Active inference rests upon the idea that the brain uses an internal generative model to predict sensory data (Parr & Friston, 2019a). These authors have argued that the brain could optimize probabilistic beliefs about external variables in a generative model such that when acting on the world, actual sensory data are consistent with the predictions inferred from the model. Inference concerning events leads to identification of putative functions of items (tool use) or of persons (interaction). Just as these processes evolve extremely rapidly, decision making also can evolve rapidly and below the level of conscious awareness (Figure 1). For example, during a go/no-go task, neural activity and behavioral measures were evident at intervals of 100 to 200 ms. and 350 to 500 ms. after stimulus presentation, corresponding to the time points of sensory evidence accumulation and decision making (Tovar et al., 2020).

Errors in prediction are the prerequisite for feedback modification of held beliefs by new information, according to the dopamine-mediated integrative action of the basal ganglia allowing for reinforcement learning (Seitz et al., 2019). Thus, beliefs can be re-enforced by the pleasure that the predicted and actual information match, whereas they may become modified when divergent information indicates a mismatch (Kraus, 2020). Moreover, these subconsciously evolving processes lead to probabilistic neural representations that allow individuals to develop preferences, maintain positions, and to tailor behavior. These processes involve integration of cognitive and emotional processes for which the anterior cingulate has been shown to be a critical hub in prefrontal circuits (Joyce et al., 2020). Furthermore, the limbic system was shown to play a differential role in this context with experienced value being encoded in the amygdala, while merit was related to anterior insula activity (Vestergaard & Schultz, 2020).

Whereas most information processing in the brain occurs outside of conscious awareness, people can recall perceived events and the predictions provided by their primal and conceptual beliefs (Scoboria et al., 2004). The conscious access to internally available information has been shown to rely on long-distance cerebral connectivity (Berkovitch et al., 2021). Their contents can be expressed verbally. People can phrase the content of what they believe in semantic terms, covertly in their mind, and overtly for communication with other people (Oakley & Halligan, 2017; Seitz & Angel, 2020). Moreover, the proposition associated with such an account can be evaluated in terms of trustworthiness, plausibility and personal relevance, which are prerequisites for belief acceptance (Connors & Halligan, 2020; Scoboria et al., 2004; Sugiura et al., 2015). Thereafter, the verbal expression of such a belief may also be stated aloud, so that others can hear it in the sense of external broadcasting (Oakley & Halligan, 2017). This flow of information, however, is not unidirectional; it is reciprocal. For instance, verbal expression of one’s thoughts may help someone elaborate his or her propositions and clarify what he or she has in mind. In fact, there is evidence that semantic processing in the left-hemispheric speech-related cortical areas can generate a linguistic content that goes beyond the original exogenous information (Kaufeld et al., 2020; Lange et al., 2013). Thus, so-called language areas are part of more extensive cortical networks that have been described to be related to believing and beliefs (Han et al., 2017; Howlett & Paulus, 2015). For that reason, many people possibly talk to themselves, assuming that a putative counterpart becomes aware of their possibly varied thoughts and beliefs. Likewise, awareness of a belief-based behavior in addition to a corresponding prediction may influence the person’s choice, e.g., that it is behaviorally relevant. For example, a person may decide to withhold a certain action or narrative, and instead replace it with another action upon contradictory social feedback (Scoboria et al., 2018).

It should be emphasized that neither the processes of believing nor beliefs are directly accessible. Rather, they are post-hoc explanatory attributions that are inferred by an observer (oneself as well as others) from what an individual states and from his/her behavior. This notion accords with the dual faced attribution theory (Malle & Korman, 2022) but applies also to many other terms that cannot be translated into a field of empirical research unless they have been operationalized sufficiently. For example, the naturalistic approach here described does not relate a person’s belief to the notion of propositional knowledge, which in philosophy has been defined as justified true belief (Lemos, 2007). According to such a definition, a proposition is true if and only if it corresponds to the facts (Bondy, 2015). This certainly pertains to objects as they are identified with high probability by other people in an identical or closely corresponding way. In fact, the corresponding statements reside on the criteria of objectivity and the perceptions may be taken as accurate and true (Leitgeb, 2017). But events and narratives change by definition, rendering their descriptions subjective and/or elusive.

## Characteristics of Memories

Memory is a single term, but it refers to a multitude of human capacities and cognitive sub-functions (Bernecker, 2009; Markowitsch, 2013). Foremost, it enables our capacity to encode information, store it, and at a later point in time to retrieve the information that was acquired previously. Moreover, it comprises different functions that are mediated by different brain structures. Most basically, memory has been dichotomized into short-term and long-term memory. Short-term memory involves the retention of pieces of information (memory chunks) for a relatively short period of time (usually up to 30 s.), whereas long-term memory can hold an indefinite amount of information for an indefinite time (Jawabri & Cascella, 2022). Long-term memory consists of declarative or procedural or memory (Squire, 1986). Declarative memory is explicit and accessible to conscious awareness concerning facts, episodes, lists, and routes of everyday life. By contrast, procedural memory is implicit and accessible only through performance by engaging skills or operations in which the knowledge (learned rules) of the performance instructions are embedded (Willingham, 1998; Willingham et al. 2002).

### Short-Term and Working Memory

In short-term memory, information can be maintained via short-term synaptic plasticity (Masse et al., 2020). Short-term memory exhibits a temporal decay of the memory content and a limited chunk capacity. A special case of short-term memory is working memory. It refers to the temporary storage of information in connection with the performance of other cognitive tasks such as reading, problem-solving, or learning (Baddeley, 1983). Thus, working memory involves manipulation of information by use of other cognitive systems including perception and action as well as attention, speech perception, and speech production (Cowan, 2008). Working memory has the ability to probabilistically associate converging information of objects and to represent an item in multiple codes (Postle, 2006). However, despite the unimodal channel by which the object may have been perceived, working memory facilitates one’s ability to manipulate or transform the representation of this information. Also, it affords the retention of information in conscious awareness when this information is not present in the environment, to its manipulation, and to its use in prospective motor coding (Postle, 2006). Because the central executive is assumed to function like a limited-capacity attentional system capable of selecting and operating control processes and strategies, reasoning and action generation have been found to take longer as the memory load increases (Baddeley, 1983). For example, a brief story, a chapter in a book, or an entire book scale the demand on working memory differently. Concerning the neural structures that are involved, there is a broad engagement of a large array of brain areas that participate in working memory tasks as summarized recently (Christophel et al., 2017). Thus, storage of working memory contents for the transformation of sensory information into a delayed behavioral response takes place in multiple regions—from sensory cortices to parietal and prefrontal areas involving distributed representations with different levels of abstractness and generalizability (Christophel et al., 2017).

### Long-Term Memory

Declarative memory comprises the so-called episodic memory of what one has personally experienced involving the conscious recollection of an episode or of the sequence of events (Bernecker, 2009). Thus, episodic memory has an autobiographical reference and, thereby, a first-person perspective. A second type of declarative memory is called propositional memory, which is largely synonymous to “semantic memory”. It represents the conscious and intentional memory of concepts and meanings (Jawabri & Cascella, 2022). Experiential and propositional memory have in common that they seek to represent the world and that their contents can in principle be articulated (Bernecker, 2009). However, the information is said to be stored in different systems, with the episodic and spatial memory encoded via the right mediotemporal formation, whereas semantic memory involves the left mediotemporal formation.

Encoding of information in long-term memory depends critically on medial temporal lobe structures (Squire & Zola-Morgan, 1988) but cortical structures are involved as well. For example, the middle frontal gyrus was shown by functional imaging to be more active at encoding for imagined words, whereas the inferior frontal gyrus was more active at encoding heard words (Sugimori et al., 2014). Moreover, a limited detectability of objects and events in the environment has been shown to impair reality filtering by the orbitofrontal cortex, which affects memory consolidation in the hippocampus (Liverani et al., 2015). Interestingly, declarative memory was shown to benefit from slow wave sleep–associated system consolidation (Diekelmann & Born, 2010).

Retrieving information from memory or remembering means to call back into conscious awareness what was experienced previously. In terms of cerebral structures involved in memory retrieval, there is the notion that the left prefrontal cortical areas were more involved in retrieving information from semantic memory, whereas right prefrontal cortex was more involved in episodic memory retrieval (Tulving et al., 1994). Within left prefrontal cortex episodic success activity was greater in ventrolateral parts, and retrieval success activity was greater in dorsolateral and anterior prefrontal cortex (Prince et al., 2005). Success of episodic memory retrieval involved particularly the posterior parahippocampal cortex and hippocampus (Prince et al., 2005), indicating an interaction of the prefrontal cortex and hippocampus formation for memory retrieval. Conversely, lesions in the anterior medial orbitofrontal cortex, basal forebrain, amygdala, and perirhinal cortex result in the temporal confusion of events (Schnider & Ptak, 1999). Furthermore, there are capacity limits in cognition which require that valuable information is prioritized for memory encoding and later retrieval (Elliott et al., 2020). In a large sample of 205 healthy subjects, multiple cognitive ability measures showed that episodic memory ability but not working memory capacity was predictive of value-directed remembering. Thus, motivational forces such as those noted above are able to influence what we remember and what we believe out of our varied life experiences.

### Memory Functions in Believing

**Figure 2:**
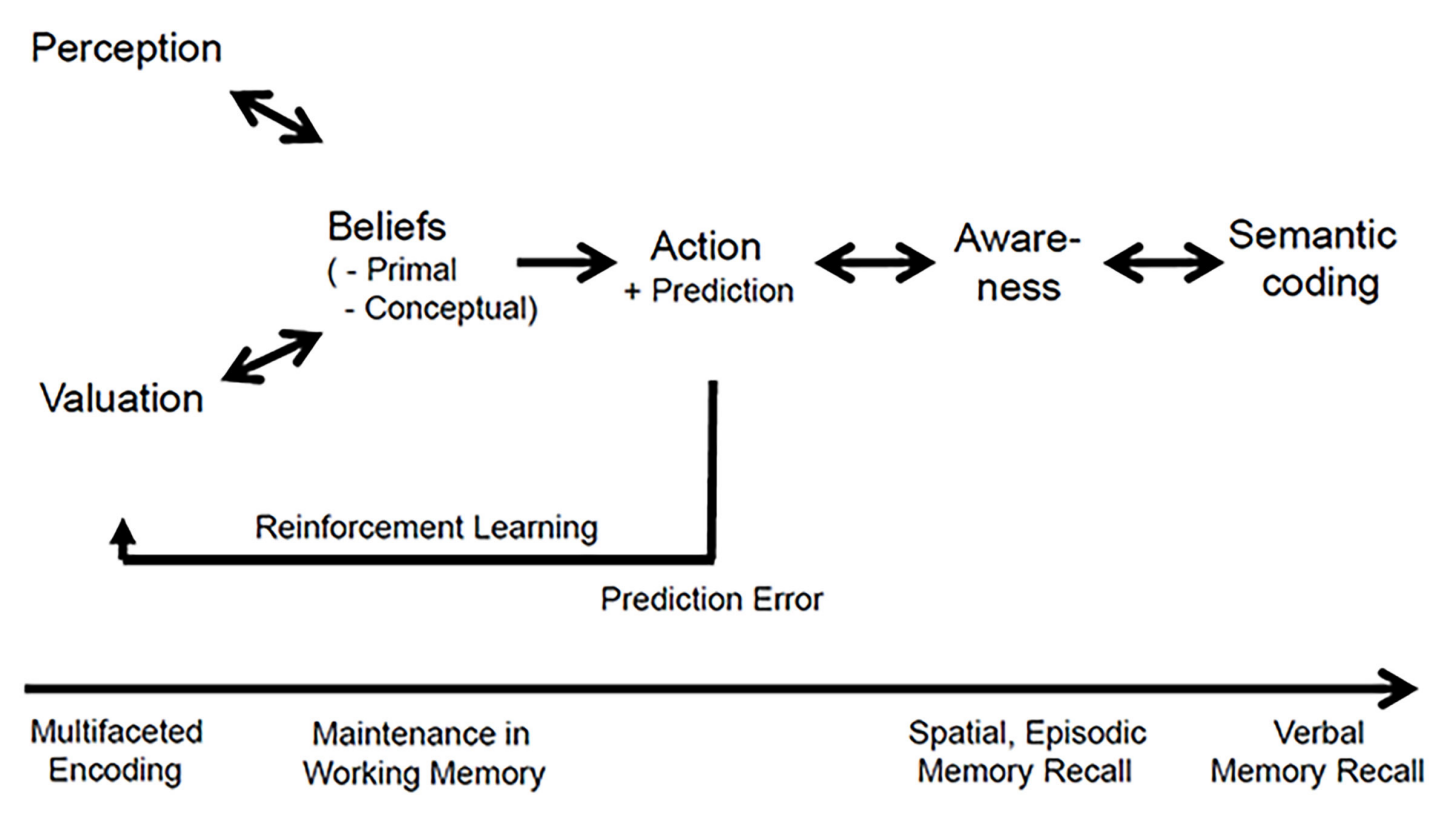
Schematic Model of the Evolution of Processes of Believing and the Interrelations With Memory Functions *Note*. Flow of information concerning awareness and semantic coding is bi-directional.

In as much as believing results from sensory perception, what is believed concerns objects and events that a person has dealt with at previous points in time (Seitz & Angel, 2020). We show here that working memory allowing us to keep the sequence of events in conscious awareness accommodates inferential reasoning about the putative cause and impact of the event (Figure 2). Moreover, the entire event including its associated emotional value is encoded in so-called episodic and autobiographic memory (Donato et al., 2021). In addition, it has been shown that emotional cues enhance memory performance such that emotional events are also more vividly remembered than neutral events (Hostler et al., 2018; Kensinger & Ford, 2020). This phenomenon is exaggerated in posttraumatic distress disorder where the frightening or aversive emotional loading of memories shakes the afflicted individual upon symptom provocation (Sartory et al., 2013). But, the inherently incomplete perception of objects and events due to a poor signal-to-noise ratio or a short observation period and the positive or negative emotional loadings of such objects and events can result in distorted beliefs and memories. Moreover, brain diseases can induce distorted beliefs of various types (Seitz, 2022). Nonetheless, a person’s beliefs of highly emotional perceptions may be modified at a later point in time by plausibility considerations that are imposed typically by other people (Mazzoni et al., 2010). We suggest that in such situations a primal belief is evaluated in terms of conceptual plausibility on the basis of comments provided by other people (Coltheart et al., 2011; Sugiura et al., 2015).

In narratives, the demands on verbal working memory may be higher or lower depending on the complexity of the transmitted information. We propose here that the emotional component influences how deeply perceived narratives are encoded in semantic long-term memory (Figure 2). Most likely, the situational circumstances are relevant when, for example, the narratives are heard and the contents of the narratives are encoded in parallel in the episodic and semantic channels. Because both aspects may be encoded with different weights, they later may be recalled differently. Nevertheless, the recall or retrieval of such encoded information is typically incomplete and, thus, probabilistic (Figure 2). It is well known that the longer the interval between the encoding and retrieval, the lesser the correspondence of the encoded and retrieved information. Likewise, a greater complexity of the information impairs the success of retrieval. These limitations of memory encoding and retrieval determine the trust of an individual into the beliefs he or she holds. In consequence, prior beliefs may faint and be replaced by new more vivid beliefs.

Notably, owing to the many repetitions, narratives about one’s origin, the society and religion build up the composition of conceptual beliefs provided by the individuals’ autobiographical memory (Fivush et al., 2011). Furthermore, owing to their memory contents they also gain normative values to the individuals (Bordalo et al., 2021). Thus, episodic autobiographical memory is considered as a pure human form of memory that is dependent on a proper ontogenetic development and shaped continuously by the social environment including culture (Markowitsch, 2013).

## Discussion

Believing and memory are fundamental and complex brain functions mediated by dedicated neural systems. We have shown here that they are interrelated in a multiple fashion. Owing to the encoding function of memory believing would stabilize a given perception in the light of its adaptive value to a given subject (Connors & Halligan, 2015). This will result in an increased effectiveness and efficiency of brain mechanisms involved in problem solving, decision making, goal setting, and in maneuvering in the environment (Connors & Halligan, 2020; Garcés & Finkel, 2019). However, owing to reinforcement learning the resulting beliefs are up-dated by new information. Moreover, believing involves working memory for inferential abstraction concerning the multimodal properties of objects, the relation of changes constituting events, and the conceptual impact of narratives. The inferential abstract information, which is loaded with subjective relevance, becomes encoded in long-term memory. This automatic encoding process is probably enhanced by repetitive encounters of the given subject with the environmental items. The memory contents can be retrieved at a later point in time from memory in a probabilistic fashion. The link between perception and semantic memory has been proposed to represent embodied cognition (Reilly et al., 2016). We have outlined here that the neural representations resulting from the processes of believing are stored in the human brain in a multiple fashion. This accords well with the notion that neural representations are memories that are localized in neural networks that encode information and, when activated, enable access to this stored information (Wood & Grafman, 2003).

As humans are in constant motion and the environment is constantly changing as was highlighted recently by Haye and Torres-Sahli (2017), we suggest that humans are in need of stable representations of their environment on which they can rely. For example, narratives help to structure time into units such as a minute, hour, week, month, and year, although we remain unable to define time (Oestreicher, 2012). Thereby, events can be defined with a beginning and an end (Zacks & Tversky, 2001), which can be determined objectively. The duration of such an event determines the amount of information characterizing the event with longer events having a higher demand on working memory. This pertains to primal beliefs that concern concrete objects and events whose perception does not involve language functions. The so-called primal world beliefs correspond, however in essence, to language-based conceptual beliefs that can be assessed with an inventory based on tweets and historical texts (Clifton et al., 2019). While primal beliefs refer to experiences that one has made previously and not to something to happen in the future, the Christian belief that the last judgment is going to take place in the future is a conceptual belief, namely the conceptual belief of the last judgment to come.

People can express their belief-based propositions, act upon them, discuss them, and evaluate them (Figure 2). Humans stand alone in their ability to understand others’ attitudes and behavioral expressions—a capacity that allows us to put ourselves in others’ shoes (Hall & Brosnan, 2017). Nevertheless, a proposition as stated may not reflect the someone’s belief or intention. This may occur because the person may refuse telling what she or he is actually believing but instead may intend to deceive the listener. Moreover, the person may have realized that the previously held belief is not plausible in view of the verbal feedback from other people (Mazzoni et al., 2010; Scoboria et al., 2004). Even self-deception that results in a false belief that is then communicated to others may occur. None the least, deficits of multiple memory-related processes lead to confabulation, which has to be differentiated from delusions that are associated with aberrant cognitive schema structure and distorted belief monitoring (Gilboa, 2010).

The cultural brain hypothesis posits that humans have the ability to store and manage information acquired through social rewards and punishment as well as through asocial or social learning (Muthukrishna et al., 2018; Williams, 2021). Recent evidence from findings in systems neuroscience and neuroimaging showed that emotion and cognition are deeply interwoven in the brain. Putatively emotional and cognitive regions dynamically influence one another via a complex web of recurrent, often indirect anatomical connections in ways that jointly contribute to adaptive behavior (Okon-Singer et al., 2015). For example, cognitive perspective taking is the ability to infer an agent’s thoughts or beliefs, while affective perspective-taking is the ability to infer an agent’s feelings and emotions (Healey & Grossman, 2018). In accordance with the claim of social neuroscience neurophysiological studies of individuals can open a platform to understand larger groups of subjects and even societies (Vogeley & Roepstorff, 2009). In particular, cultural information can provide an important influence of emotions in the mutual constitution of social reality and shared reality (Kashima et al., 2018). Accordingly, validation of religious beliefs has been reported to occur by ritually addressing the emotions of the participants (Atran & Norenzayan, 2004).

Here, we may touch upon related topics which have recently attracted scientific interest such as the notion of extended cognition (Sprevak, 2019) or collective intentionality. While intentionality refers to the capacity of mental states to be about or directed towards an object or state, collective intentionality refers to the possibility that groups themselves are the bearers of mental states (Tollefsen & Friedlaender, 2017). In anthropological studies the role of cultural memory and its impact on societies has been advanced since the milestone work *On Collective Memory* (Halbwachs, 1992). This notion sheds specific light on the history of culture since Antiquity (J. Assmann, 2012). It highlights the role of cultural memory for modern Western societies (A. Assmann, 2012) and their formation and maintenance of identity (J. Assmann & Czaplicka, 1995). It even appears to provide a link to the topic of religions and their evolution(s) that can be understood in relation to the evolution of religiosity and the capacity of believing (Angel, 2020). We suggest that semantic memory is a most important determinant of the multifaceted memory representations of beliefs for the evolution of individuals in society. Finally, we emphasize that in contrast to the believing and the memory functions dealt with here, beliefs and memories are post-hoc attributions inferred from a third person perspective concerning behavior observed in individuals (Parr & Friston, 2019b). Consequently, memories and beliefs are comparable to other attributions such as knowledge, justification, motives, goals, and the social content (Johnson et al., 2012). Also, in this perspective the propositions starting with “I believe …” imply meta-cognition, which is based on an individual’s inferential memory recall.

### Conclusion

Believing is a fundamental brain function resulting in probabilistic, personally meaningful representations of external information about objects, events and narratives that can be termed beliefs. Owing to the intricate relation of believing to memory functions, beliefs can be maintained in the brain and determine a person’s behavior over extended periods of time. Thus, believing and beliefs that are the essentials of the concept of creditions have broad implications for social life. They open novel perspectives for cognitive and social psychology as we describe in a forthcoming paper.
